# Single Strain Isolation Method for Cell Culture-Adapted Hepatitis C Virus by End-Point Dilution and Infection

**DOI:** 10.1371/journal.pone.0098168

**Published:** 2014-05-21

**Authors:** Nao Sugiyama, Asako Murayama, Ryosuke Suzuki, Noriyuki Watanabe, Masaaki Shiina, T. Jake Liang, Takaji Wakita, Takanobu Kato

**Affiliations:** 1 Department of Virology II, National Institute of Infectious Diseases, Tokyo, Japan; 2 Department of Gastroenterology and Hepatology, Shin-Yurigaoka General Hospital, Kawasaki, Kanagawa, Japan; 3 Liver Diseases Branch, National Institute of Diabetes and Digestive and Kidney Diseases, National Institutes of Health, Bethesda, Maryland, United States of America; Inserm, U1052, UMR 5286, France

## Abstract

The hepatitis C virus (HCV) culture system has enabled us to clarify the HCV life cycle and essential host factors for propagation. However, the virus production level of wild-type JFH-1 (JFH-1/wt) is limited, and this leads to difficulties in performing experiments that require higher viral concentrations. As the cell culture-adapted JFH-1 has been reported to have robust virus production, some mutations in the viral genome may play a role in the efficiency of virus production. In this study, we obtained cell culture-adapted virus by passage of full-length JFH-1 RNA-transfected Huh-7.5.1 cells. The obtained virus produced 3 log-fold more progeny viruses as compared with JFH-1/wt. Several mutations were identified as being responsible for robust virus production, but, on reverse-genetics analysis, the production levels of JFH-1 with these mutations did not reach the level of cell culture-adapted virus. By using the single strain isolation method by end-point dilution and infection, we isolated two strains with additional mutations, and found that these strains have the ability to produce more progeny viruses. On reverse-genetics analysis, the strains with these additional mutations were able to produce robust progeny viruses at comparable levels as cell culture-adapted JFH-1 virus. The strategy used in this study will be useful for identifying strains with unique characteristics, such as robust virus production, from a diverse population, and for determining the responsible mutations for these characteristics.

## Introduction

Hepatitis C virus (HCV) is one of the most important pathogens causing liver-related morbidity and mortality [1], [2]. HCV is a positive-stranded RNA virus belonging to the Flaviviridae family. Its genome, about 9.6-kb long, consists of an open reading frame (ORF) encoding a large polyprotein that is cleaved by cellular and viral proteases into at least 10 structural and non-structural (NS) proteins [3], [4]. The structural proteins include core, E1 and E2, which form virus particles. The NS proteins include p7, NS2, NS3, NS4A, NS4B, NS5A and NS5B, which are associated with viral replication.

For research into the HCV life cycle and development of antivirals, *in vitro* models of this virus are indispensable. First, an HCV subgenomic replicon system was used to examine HCV replication in cell culture [5], [6]. The HCV infectious step has been assessed by an HCV pseudo-particle (HCVpp) system harboring E1 and E2 glycoproteins [7], [8]. This system enabled us to identify several HCV receptors. Finally, to investigate other steps in the HCV life cycle, an HCV cell culture system was developed with a unique genotype 2a strain, JFH-1 [9]. This strain is able to replicate efficiently in culture cells, and its characteristics enabled us to observe the whole life cycle of this virus in cell culture by using cell-culture generated HCV (HCVcc) [10]–[12].

By modifying this system with CD81-lacking HuH-7-derived cells, we established a novel system designated the single cycle virus production assay, and this enabled us to estimate the efficiency of each step of viral replication, infectious virus production, secretion and infection [13]–[16]. However, virus production levels of wild-type JFH-1 (JFH-1/wt) in these systems are limited, and this shortage sometimes leads to difficulties in experiments that require high viral concentrations. To overcome these shortcomings, recent studies have identified several adaptive or compensatory mutations that enhance viral production of JFH-1 [17]–[24]. The contributions of these mutations to the viral life cycle are not well defined. In this study, we isolated the cell culture-adapted JFH-1 virus, which that can efficiently produce progeny viruses by serial passaging of JFH-1 transfected Huh-7.5.1 cells, and evaluated the affected steps in the viral life cycle.

## Materials and Methods

### Cell Culture

The HuH-7-derived cell lines Huh-7.5.1, provided by Francis Chisari (Scripps Research Institute, La Jolla, CA), and Huh7-25, which lacks CD81 expression, were cultured at 37°C in a 5% CO2 environment using Dulbecco's Modified Eagle's Medium containing 10% fetal bovine serum [11], [25]. 293T cells were also kept under the same conditions.

### Plasmid Construction and RNA Transfection

Mutation-introduced JFH-1 variants were prepared by site-directed mutagenesis with appropriate primers. The methods of *in vitro* RNA synthesis and electroporation were described previously [26], [27].

### Quantification of HCV RNA and Core Antigen

Total RNA was extracted from 140 µL of culture medium or from harvested cell pellets, and the real-time quantitative RT-PCR was performed to determine the HCV RNA titer as described previously [28]. The concentration of total RNA in the cells was also measured. The concentration of HCV core antigen (Ag) in culture medium and cell lysates were measured by the Lumipulse Ortho HCV Ag kit (Ortho Clinical Diagnostics, Tokyo, Japan) [29].

### Titration of HCV Infectivity

The infectivity titers of HCV were measured by indirect immunostaining as described previously [27]. The infectivity titer was expressed as focus-forming units (FFU) per mL. The intracellular infectivity and specific infectivity titer were determined as described previously [14].

### HCV Pseudo-Particles Assay

HCV pseudo-particles (HCVpp) containing E1 and E2 glycoproteins of wild-type or mutation-introduced JFH-1 were produced as described previously [7], [8]. To adjust the amount of virus, copy number of packaged luciferase reporter RNA was quantified by real-time detection PCR with primers and probe as reported previously [30].

### HCV Trans-complemented Particles Assay

Generation and infection of HCV trans-complemented particles (HCVtcp) has been reported elsewhere [31]–[33]. Briefly, the RNA polymerase I-driven JFH-1 reporter replicon plasmid (pHH/SGR-Luc) and the CAG promoter-driven JFH-1 core – NS2 expression plasmid (pCAGC-NS2_JFH1) or T416N mutation in the E2 region introduced pCAGC-NS2/JFH1 (pCAGC-NS2_JFH1/T416N) were co-transfected into Huh-7.5.1 cells. Culture medium was harvested at 6 days after transfection, and was passed through a 0.45- µM pore-size filter for infection. To adjust the amount of virus, RNA in culture medium was extracted with the QIAamp Viral RNA kit, treated with DNase (TURBO DNase; Ambion, Austin, TX), and purified with an RNeasy Mini kit using on-column DNase digestion (QIAGEN). Copy number of HCV was then measured as described previously [34]. Generated viruses were infected into naïve Huh-7.5.1 cells, and cells were harvested at 72 h for analysis of luciferase activity.

### HCV Sequencing

Total RNA was extracted from culture medium, and cDNA was synthesized using Superscript III (Invitrogen, Carlsbad, CA) with random 6-mer primer. Synthesized cDNA was subsequently amplified by nested-PCR covering almost the entire open reading frame and part of the 5′-untranslated region with TaKaRa LA *Taq* DNA polymerase (Takara Bio, Shiga, Japan), as described previously [14], and the sequence of amplified fragments was determined directly.

### Density gradient analysis

The culture medium of JFH-1 and variants -transfected cells were layered on top of 10–40% iodixanol gradient and centrifuged for 16 h at 40,000 rpm, 4°C in an SW-41 rotor. Fractions were collected from the top of gradient, and the density, HCV core Ag and infectivity titer in each fraction was measured.

### Statistical Analysis

Experiments were performed in triplicate, and obtained data are expressed as means ± standard deviation. Statistical analysis was performed by Student's t-test. The *p* values of less than 0.05 are considered to be statistically significant.

## Results

### Isolation of Cell Culture-adapted JFH-1

**Figure 1:**
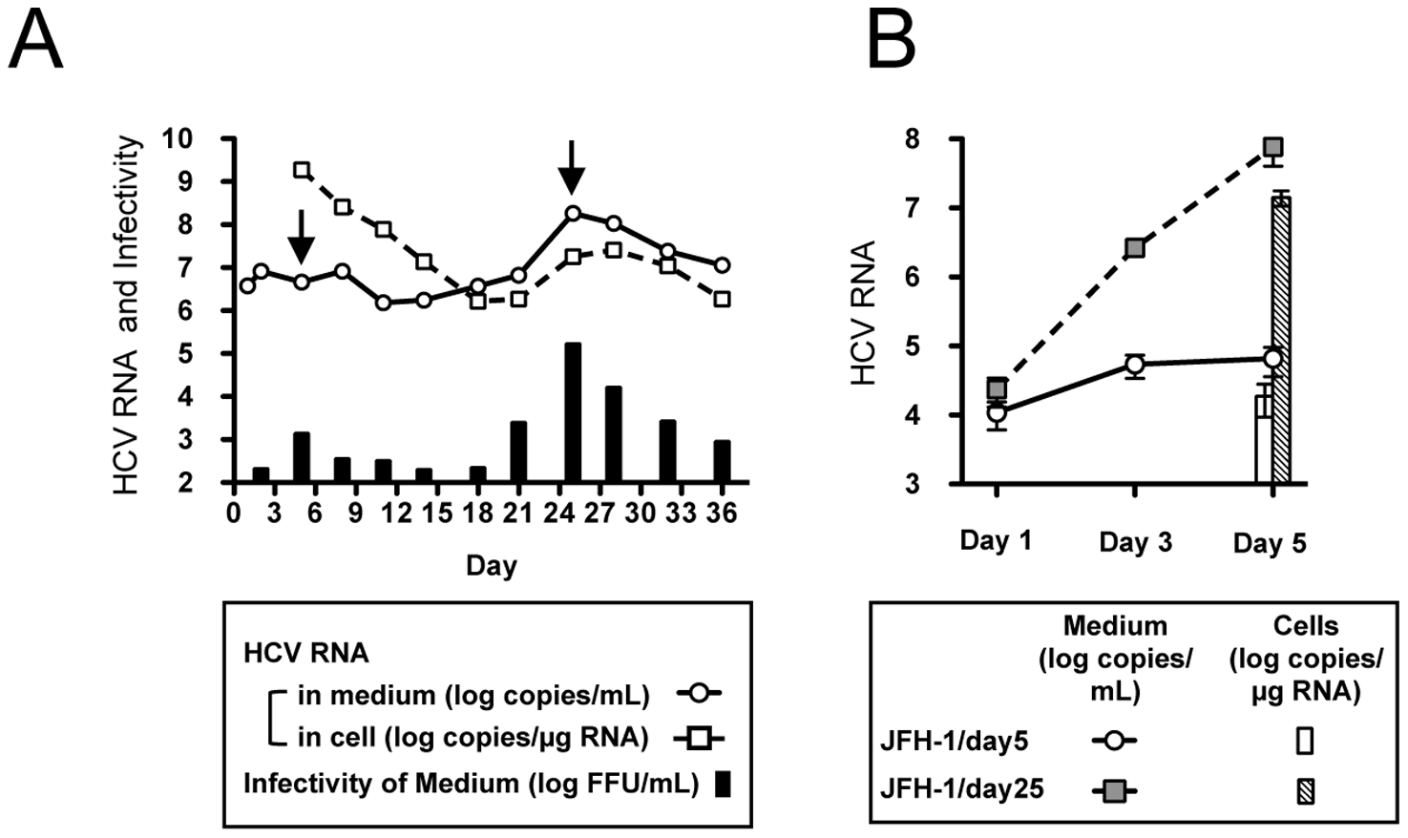
Isolation of cell culture-adapted JFH-1. (A) Long-term culture of JFH-1-transfected cells. The full-genome JFH-1 RNA was transfected into Huh-7.5.1 cells, and transfected cells were passaged for 3 to 4 days. HCV RNA titers in culture medium and cells were monitored. Arrows indicate the harvest points used for the infection study. (B) Production of progeny by infection with viruses harvested at day 5 and day 25 after transfection. The same amount of virus was used for infection at a multiplicity of infection (MOI) of 0.5, and HCV RNA titer was monitored.

In order to obtain cell culture-adapted JFH-1, we passaged full-length JFH-1 RNA-transfected Huh-7.5.1 cells and monitored extra- and intra-cellular HCV RNA and infectivity of culture medium. At 25 days after transfection, HCV RNA and infectivity titer in culture medium peaked (Figure 1A). To assess cell-culture adaptation, we compared the progeny virus production levels by infection with the same amount of viruses harvested at day 5 (JFH-1/day5) and at day 25 (JFH-1/day25). The intra- and extra-cellular HCV RNA titers of JFH-1/day25-infected cells were 1.42×10^7^±3.49×10^6^ copies/ µg RNA and 7.66×10^7^±3.61×10^7^ copies/mL, respectively, which was 3 log-fold higher than those of JFH-1/day5-infected cells (Figure 1B).

### Responsible Mutations in Cell Culture-adapted JFH-1 virus

**Figure 2:**
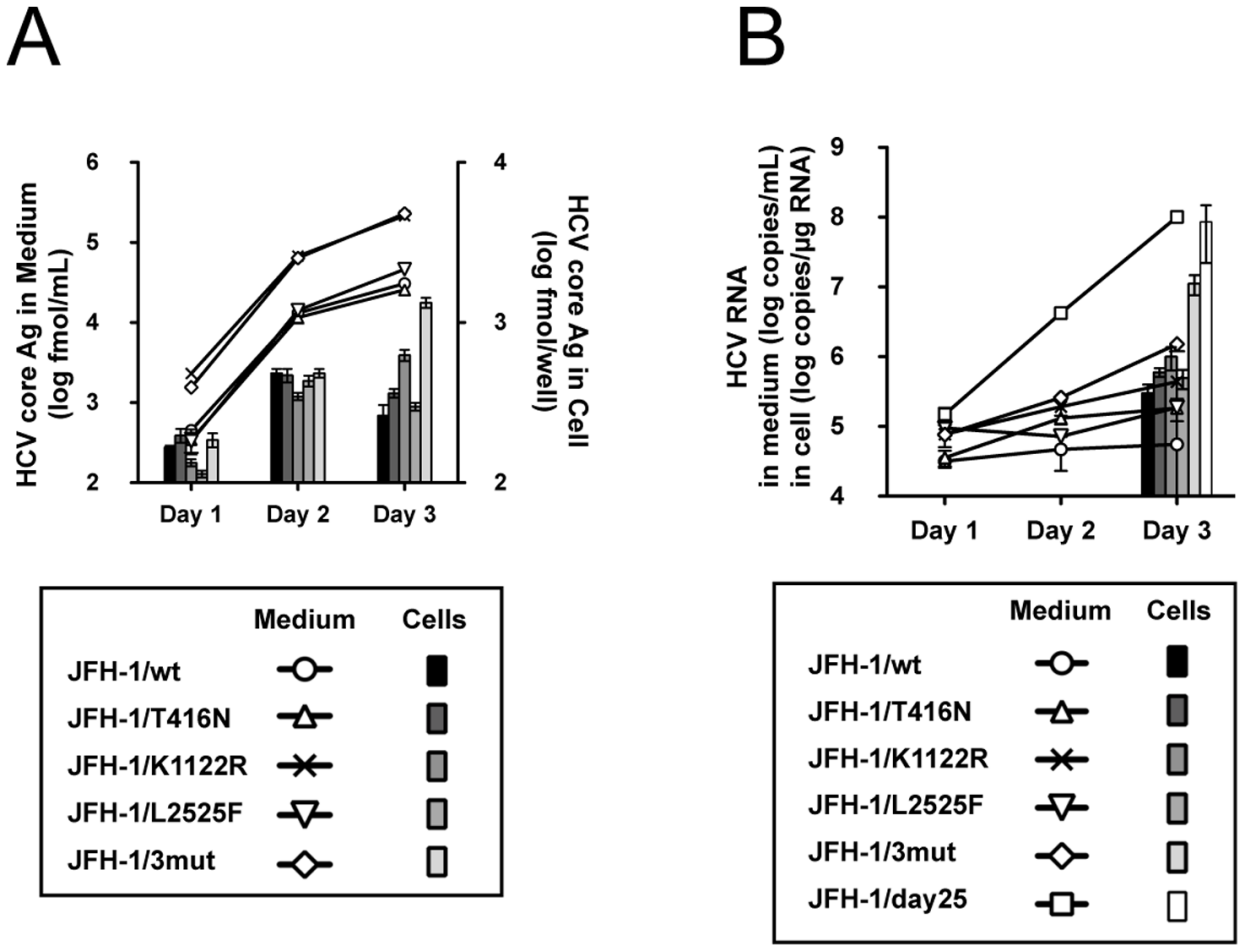
Effects of cell culture-adapted mutations on virus propagation. (A) One million cells were transfected with 2 µg of *in vitro*-transcribed RNA from JFH-1/wt, JFH-1/T416N, JFH-1/K1122R, JFH-1/L2525F and JFH-1/3mut. HCV propagation was monitored by measuring HCV core Ag. (B) The same amounts of JFH-1/wt, JFH-1/T416N, JFH-1/K1122R, JFH-1/L2525F, JFH-1/3mut and JFH-1/day25 viruses were used for infection of naïve Huh-7.5.1 cells (MOI  = 1.0), and HCV RNA titers were subsequently monitored.

In order to identify the responsible mutations introduced in the cell culture-adapted virus (JFH-1/day25), we directly sequenced the virus ORF. As indicated in Table 1, we identified 3 non-synonymous mutations at E2 (T416N), NS3 (K1122R) and NS5B (L2525F). To assess the effects of these mutations on HCV propagation, we generated the JFH-1 full-genome constructs with these mutations solely (JFH-1/T416N, JFH-1/K1122R and JFH-1/L2525F) or in combination (JFH-1/3mut). In the transfection assay with full-length HCV RNAs transcribed from these constructs, HCV core Ag in culture medium of JFH-1/K1122R and JFH-1/3mut transfected cells was approximately 1 log-fold higher than that of JFH-1/wt and other variants transfected cells (Figure 2A). HCV core Ag in cells was highest in JFH-1/3mut RNA-transfected cells, followed by JFH-1/K1122R. In the infection study of these variants (multiplicity of infection (MOI)  = 0.1), the HCV RNA titer in culture medium of JFH-1/3mut infected cells was highest among these variants and JFH-1/wt, but was approximately 2 log-fold lower than that of cell culture-adapted JFH-1 virus, JFH-1/day25. The intra-cellular HCV RNA titer of culture-adapted JFH-1 virus infected cell was also higher than that of JFH-1/3mut or other variants infected cells (Figure 2B).

**Table 1:** Mutations Detected in Cell Culture-adapted JFH-1 Variants.

Region	Identified mutation		JFH-1/day25	2G	6B
	Nucleotide	Amino Acid^a^			
E1	C1198T	−	+	+	+
E2	C1587A	T416N	+	+	+
p7	T2612G	L758V		+	
	T2641A	H767Q			+
NS3	A3631G	−			+
	A3705G	K1122R	+	+	+
	G3715A	−			+
	A4294G	I1318M			+
	C5182T	−			+
NS5A	G7069A	−			+
	G7658C	V2440L		+	+
NS5B	C7913T	L2525F	+	+	+
	G8458C	−		+	
	C8932T	−	+	+	+
	A9235G	−	+	+	+

a ‘−’ means synonymous mutation.

**Figure 3:**
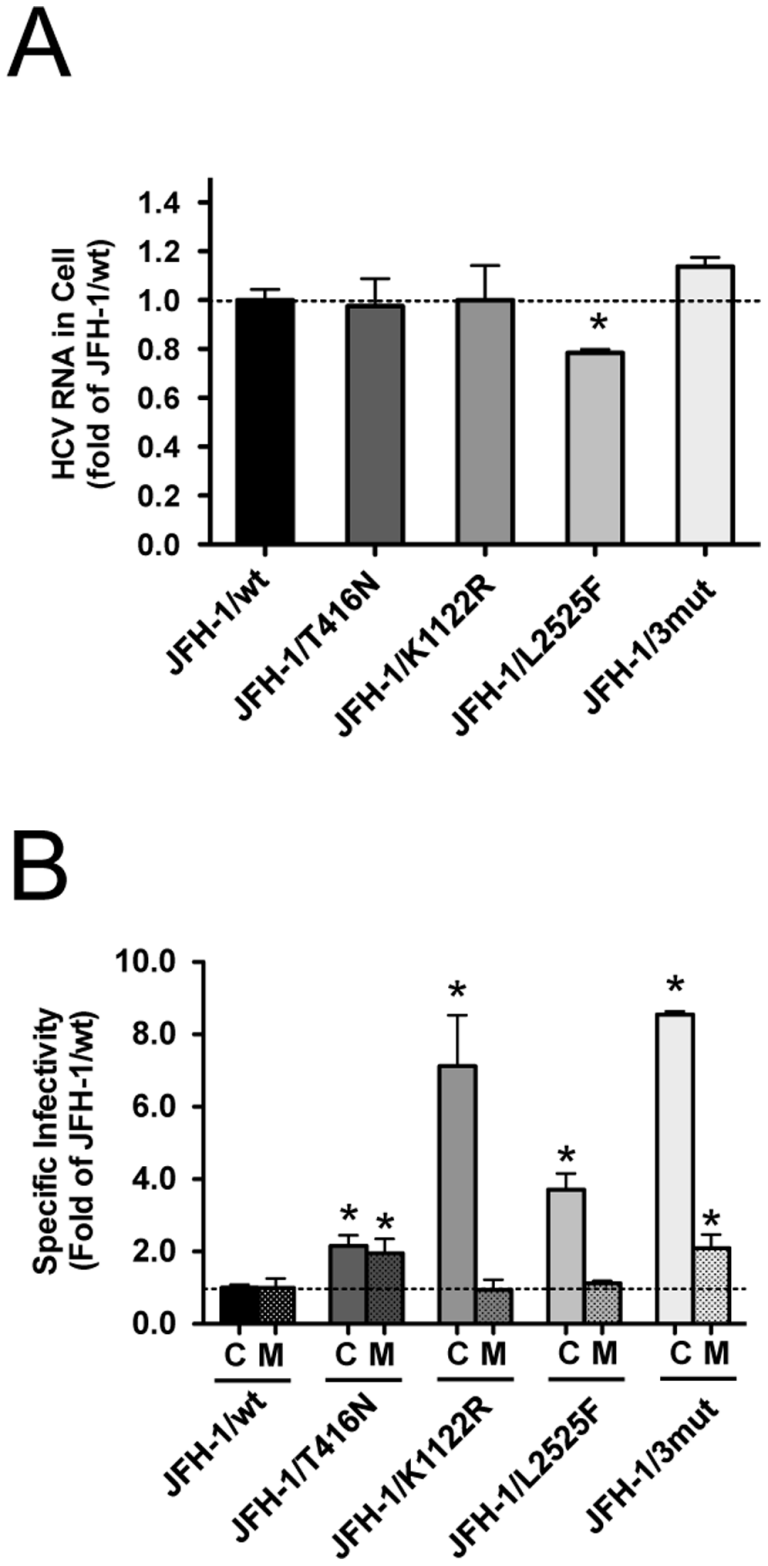
Single cycle virus production assay to assess contribution of mutations on viral life cycle. (A) Intra-cellular HCV RNA titers were assessed in full-length RNA of JFH-1/wt and its variants transfected into Huh7–25 cells. Data are given as fold change vs. JFH-1/wt. (B) Intra- and extra-cellular specific infectivity of JFH-1/wt and its variants transfected into Huh7–25 cells were calculated. Data are given as fold change vs. JFH-1/wt. C; intracellular specific infectivity, M; extracellular specific infectivity, *p<0.05.

**Figure 4:**
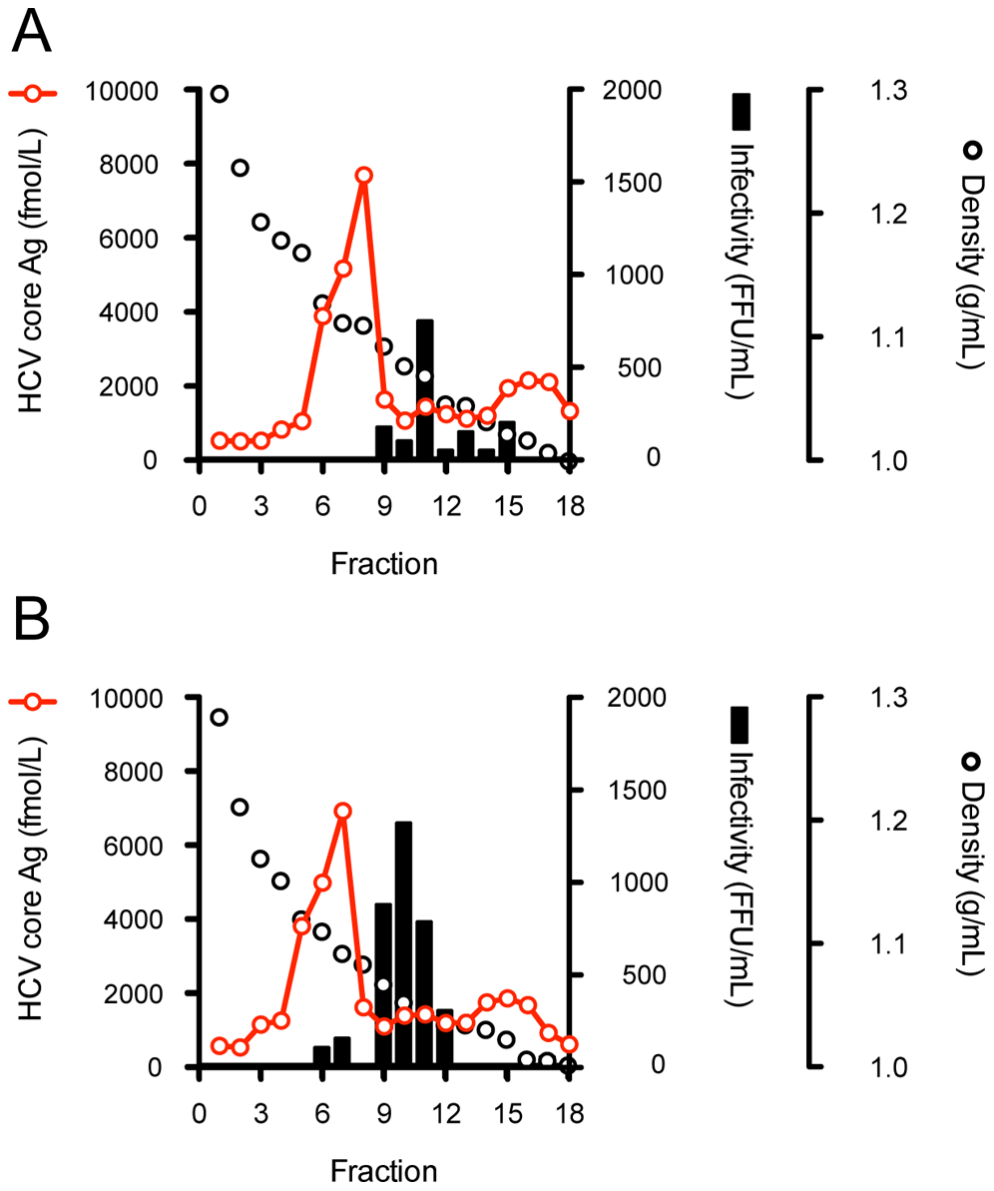
Iodixanol density gradient analysis of JFH-1/wt and JFH-1/T416N. Huh7.5.1 cells were transfected with full-length RNAs of JFH-1/wt and JFH-1/T416N. Culture medium of each strain was collected and analyzed by 10%–40% of iodixinol density gradient. Fractions were collected, and HCV core Ag and infectivity titers JFH-1/wt (A) JFH-1/T416N (B) were measured.

**Figure 5:**
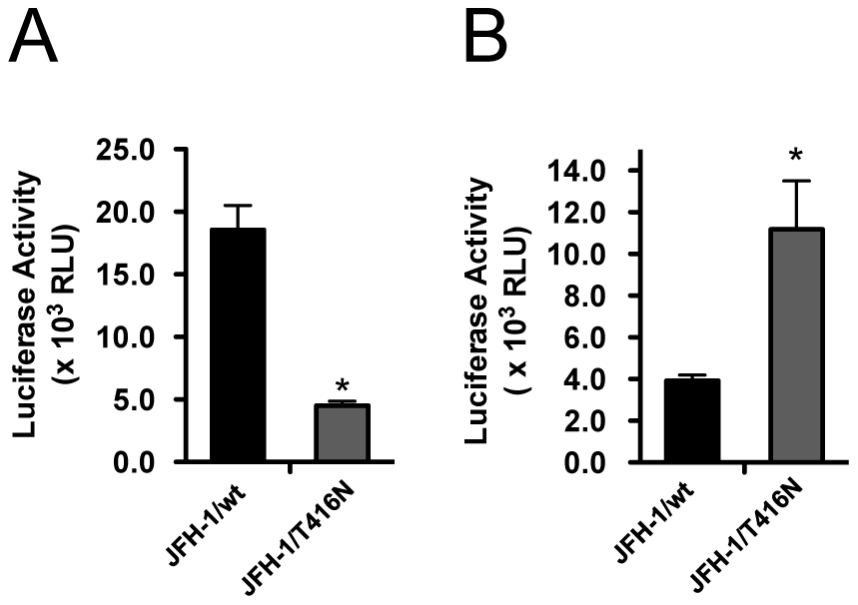
Assessment of T416N on infection step by HCVpp and HCVtcp. (A) Infectivity of JFH-1/wt and JFH-1/T416N with HCVpp envelopes was assessed. Luciferase activity was measured in HCVpp-infected cells. *p<0.05. (B) Infectivity of HCVtcp with the structural regions of JFH-1/wt and JFH-1/T416N was assessed. Luciferase activity was measured in HCVtcp-infected cells. *p<0.05.

In order to assess the function of these mutations on steps of the virus lifecycle, we used the single cycle virus production assay using Huh7–25 cells, which lacks surface expression of CD81. We compared the intra-cellular HCV RNA titer of these variants transfected Huh7–25 cells in order to assess the effects of mutations on HCV replication. The intra-cellular HCV RNA titer of JFH-1/L2525F was lower than that of other variants and JFH-1/wt (Figure 3A). To assess the effects of mutations on infectious virus production in culture-cells and the efficiency of infection, we compared the specific infectivity of these variants in transfected Huh7–25 cells. The mutations K1122R and L2525F enhanced intra-cellular specific infectivity by 7.2- and 3.7-fold, respectively, although extra-cellular specific infectivity was not affected, thus suggesting their contribution to intra-cellular infectious virus production. JFH-1/3mut containing 3 mutations also showed an 8.5-fold increase in intra-cellular specific infectivity (Figure 3B). On the other hand, JFH-1/T416N-transfected cells showed 2-fold higher intra- and extra-cellular infectivity, as compared with JFH-1/wt and other variants. JFH-1/3mut also showed enhanced extra-cellular infectivity in addition to the effects of K1122R and L2525F (Figure 3B). These data suggest that T416N enhances the infection step. To assess the biophysical properties of particles with T416N, we analyzed the culture medium of JFH-1/wt- and JFH-1/T416N- transfected cells in the density gradient. However, the density gradient profiles of these strains were similar and we could not detect the mutation specific peak of infectivity in the density gradient of JFH-1/T416N. The peak density of infectivity titer of JFH-1/T416N (1.05 g/mL) was almost identical with that of JFH-1/wt (1.07 g/mL), but the peak infectivity titer of JFH-1/T416N was 1.75-fold higher than that of JFH-1/wt (Figure 4). To confirm the advantage of T416N in the infection step, we exploited the HCVpp system. T416N was introduced into JFH-1 E1 and E2 glycoprotein-expressing vector and generated HCVpp harboring envelope proteins of JFH1/wt and JFH1/T416N. To adjust the amount of HCVpp, the copy number of packaged luciferase reporter RNA was measured. The same copy numbers of HCVpp JFH-1/wt and JFH-1/T416N were infected into naïve Huh-7.5.1 cells and luciferase activities were compared. In contrast to expectations, luciferase activity in JFH-1/T416N HCVpp-infected cells was lower than in JFH-1/wt HCVpp-infected cells (Figure 5A). We also examined the effects of T416N using the recently developed HCVtcp system. This HCVtcp contains the HCV subgenomic replicon and supports single-round infection. In contrast to the HCVpp system, we were able to observe consistent results with single cycle virus production assay in the HCVtcp system. Luciferase activity in JFH-1/T416N HCVtcp-infected cells was 2.8-fold higher than in JFH-1/wt HCVtcp-infected cells (Figure 5B).

### Single Strain Isolation Method of Cell Culture-adapted Virus by End-point Dilution and Infection

**Figure 6:**
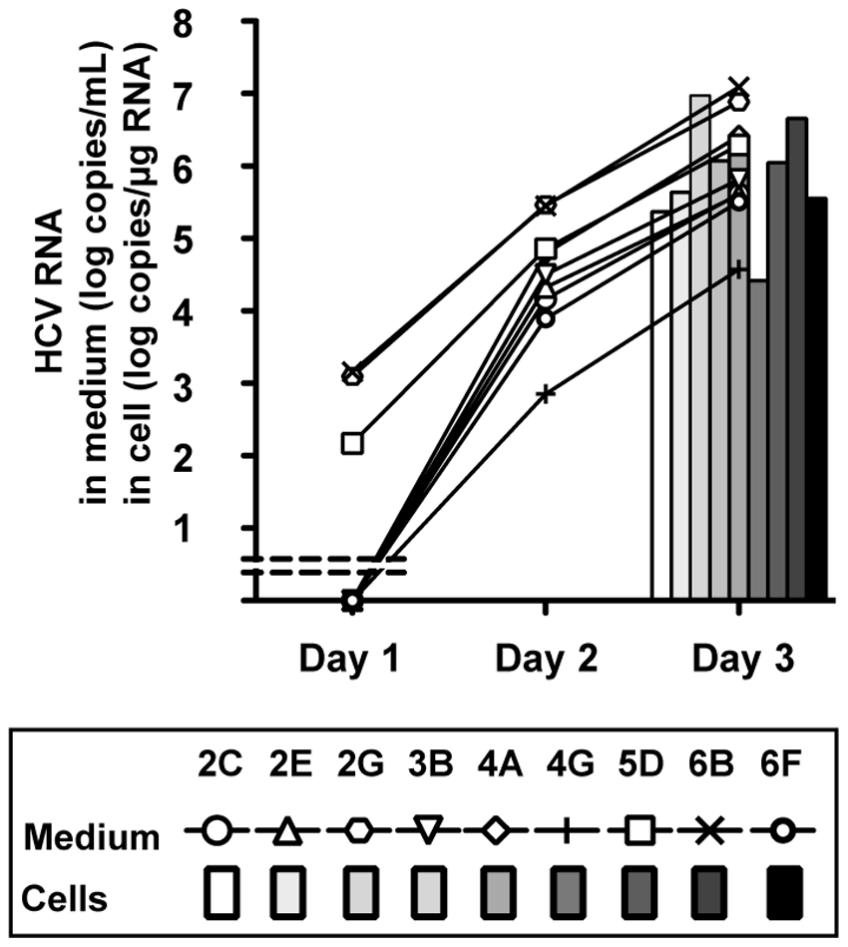
Production of progeny virus after infection of culture media harvested in wells that have a single focus. HCV RNA titers were measured in culture medium and cells.

In order to isolate the JFH-1 variants that can produce more progeny viruses, the JFH-1/day25 virus was diluted and infected into naïve Huh-7.5.1 cells seeded in a 96-well plate at a concentration of 1 FFU per well. After 72-h culture, media were kept in another plate and cells were fixed and stained with anti-HCV core antibody to visualize the foci. Culture media were then harvested from wells that contained a single focus and were used to re-infect naïve Huh-7.5.1 cells. The production of progeny viruses were compared by measuring the HCV RNA titer of infected cells (Figure 6). Inoculation with harvested media resulted in varied progeny virus production. HCV RNA titers in culture medium were 4 to 7 log copies/mL on day 3 after infection.

**Figure 7:**
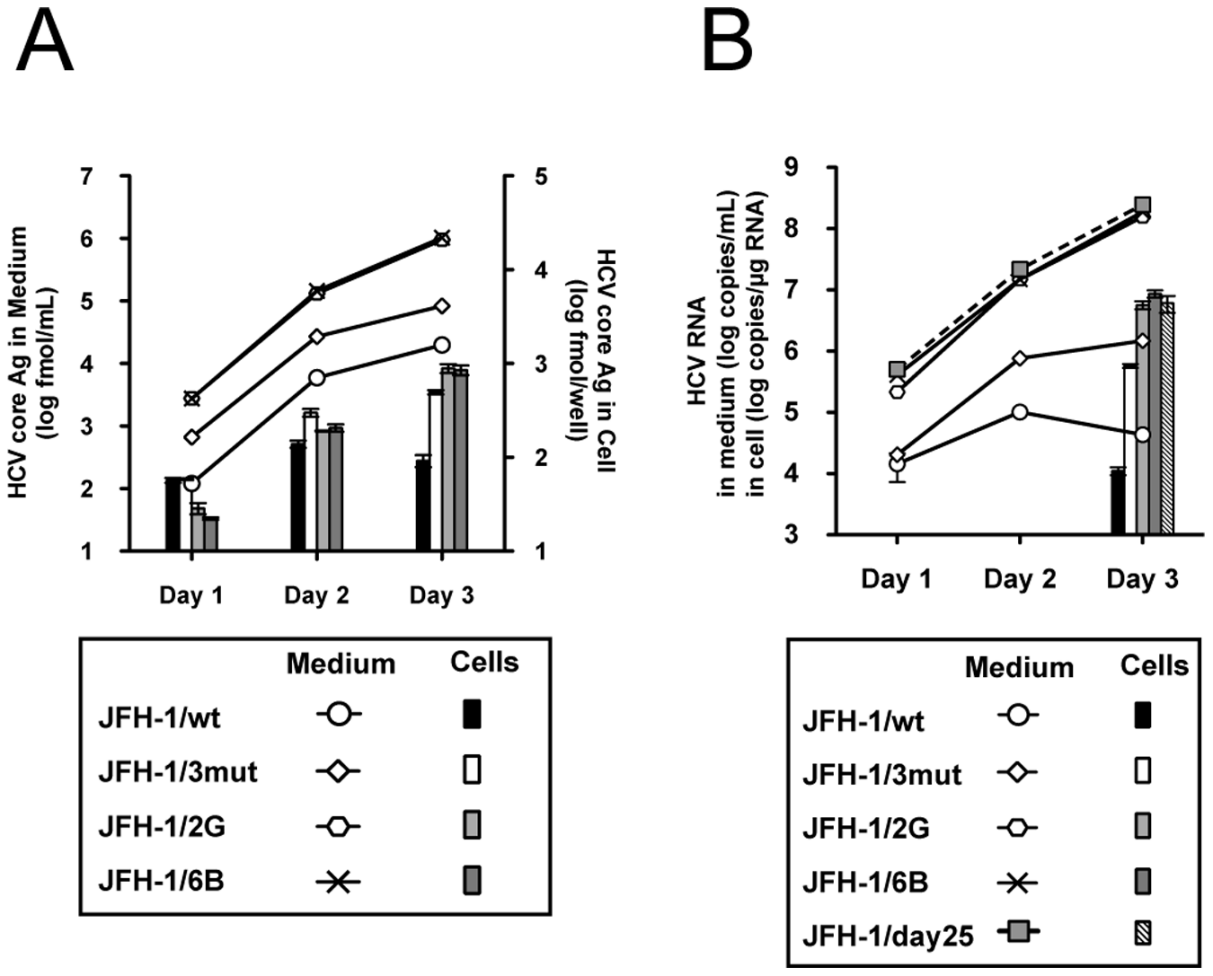
Effects of mutations detected after single-strain isolation method. (A) One million cells were transfected with 2 µg of *in vitro*-transcribed RNA of JFH-1/wt, JFH-1/3mut, JFH-1/2G and JFH-1/6B. HCV propagation was monitored by measuring HCV core Ag. (B) The same amount of JFH-1/wt, JFH-1/3mut, JFH-1/2G, JFH-1/6B and JFH-1/day25 viruses were infected into naïve Huh-7.5.1 cells (MOI  = 1.0), and HCV RNA titers were monitored.

Among the assessed variants, we selected two strains, 2G and 6B, which showed the highest virus production. Direct sequencing of these isolated strains revealed that they possessed more non-synonymous mutations, in addition to those observed in JFH-1/3mut. The 2G strain had 2 additional mutations; L758V at p7 and V2440L at NS5A. The 6B strain had 3 additional mutations; H767Q at p7, I1318M at NS3 and V2440L at NS5A (Table 1). We generated JFH-1 variants with these mutations and designated them as JFH-1/2G and JFH-1/6B. When transfected with full-genome RNA, extracellular HCV core Ag was approximately 50-fold and 10-fold higher when compared with JFH-1/wt and JFH-1/3mut, respectively, and intracellular HCV core Ag was approximately 10-fold and 2-fold higher when compared with JFH-1/wt and JFH-1/3mut, respectively (Figure 7A).

**Figure 8:**
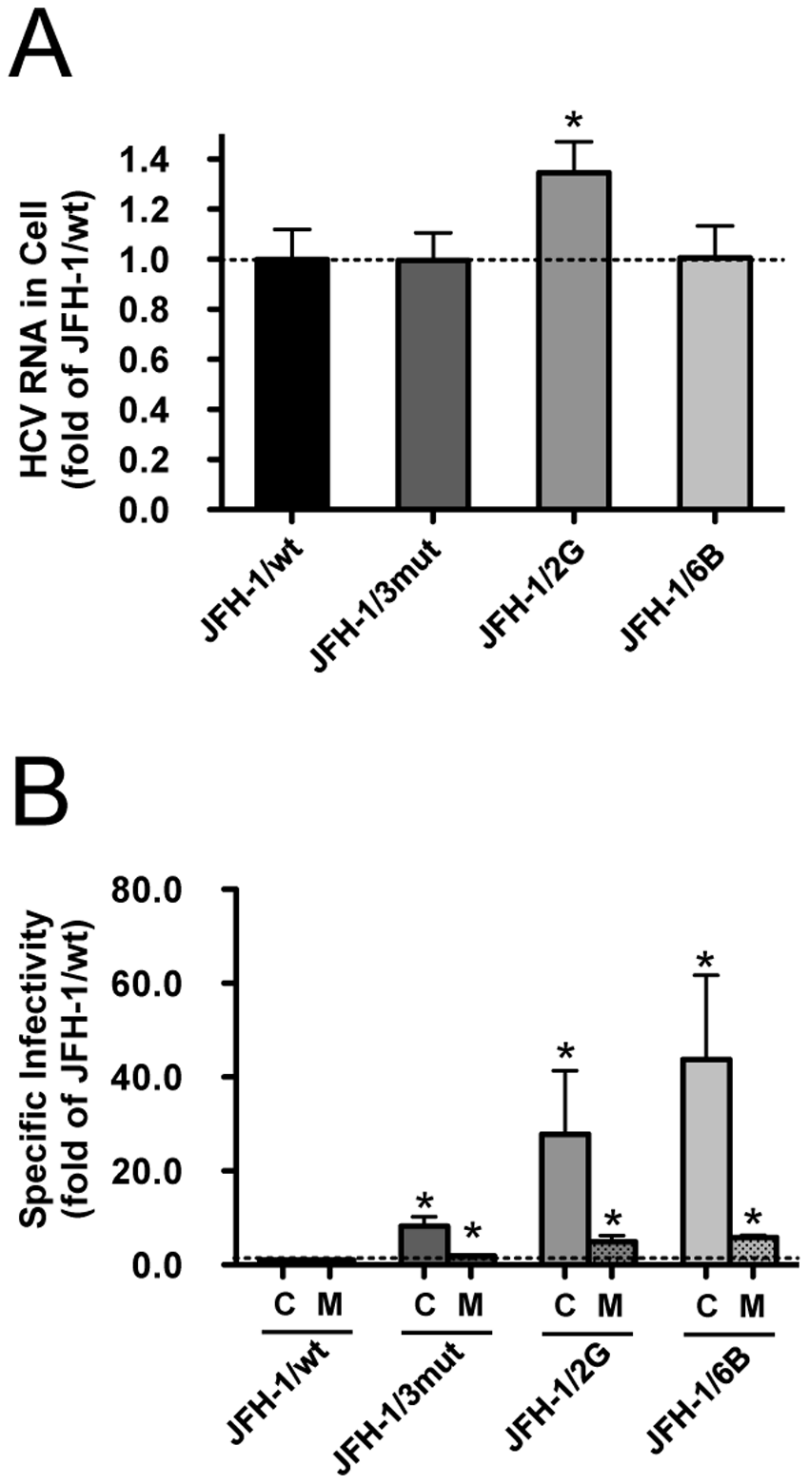
Single cycle virus production assay to assess the contribution of introduced mutations on viral life cycle. (A) Intra-cellular HCV RNA titers were assessed in full-length RNA of JFH-1/wt-, JFH-1/3mut-, JFH-1/2G- and JFH-1/6B-transfected Huh7–25 cells. Data are presented as fold change vs. JFH-1/wt. (B) Intra- and extra-cellular specific infectivity of JFH-1/wt-, JFH-1/3mut-, JFH-1/2G- and JFH-1/6B-transfected Huh7–25 cells were calculated. Data are given as fold change vs. JFH-1/wt. C; intracellular specific infectivity, M; extracellular specific infectivity, *p<0.05.

In an infection study at MOI  = 0.1, the variants JFH-1/2G and JFH-1/6B produced more progeny viruses than JFH-1/wt and JFH-1/3mut, and the production levels were comparable to that of JFH-1/day25 (Figure 7B). To assess the effects of additional introduced mutations in these variants on the virus life cycle, we used a single cycle virus production assay. After transfection of full-length RNA from JFH-1/wt, JFH-1/3mut, JFH-1/2G and JFH-1/6B into Huh7–25 cells, the intra-cellular HCV RNA titer was compared. We found that JFH-1/2G-transfected cells showed 1.35-fold higher intracellular HCV RNA titer, thus suggesting enhanced viral replication (Figure 8A).

**Figure 9:**
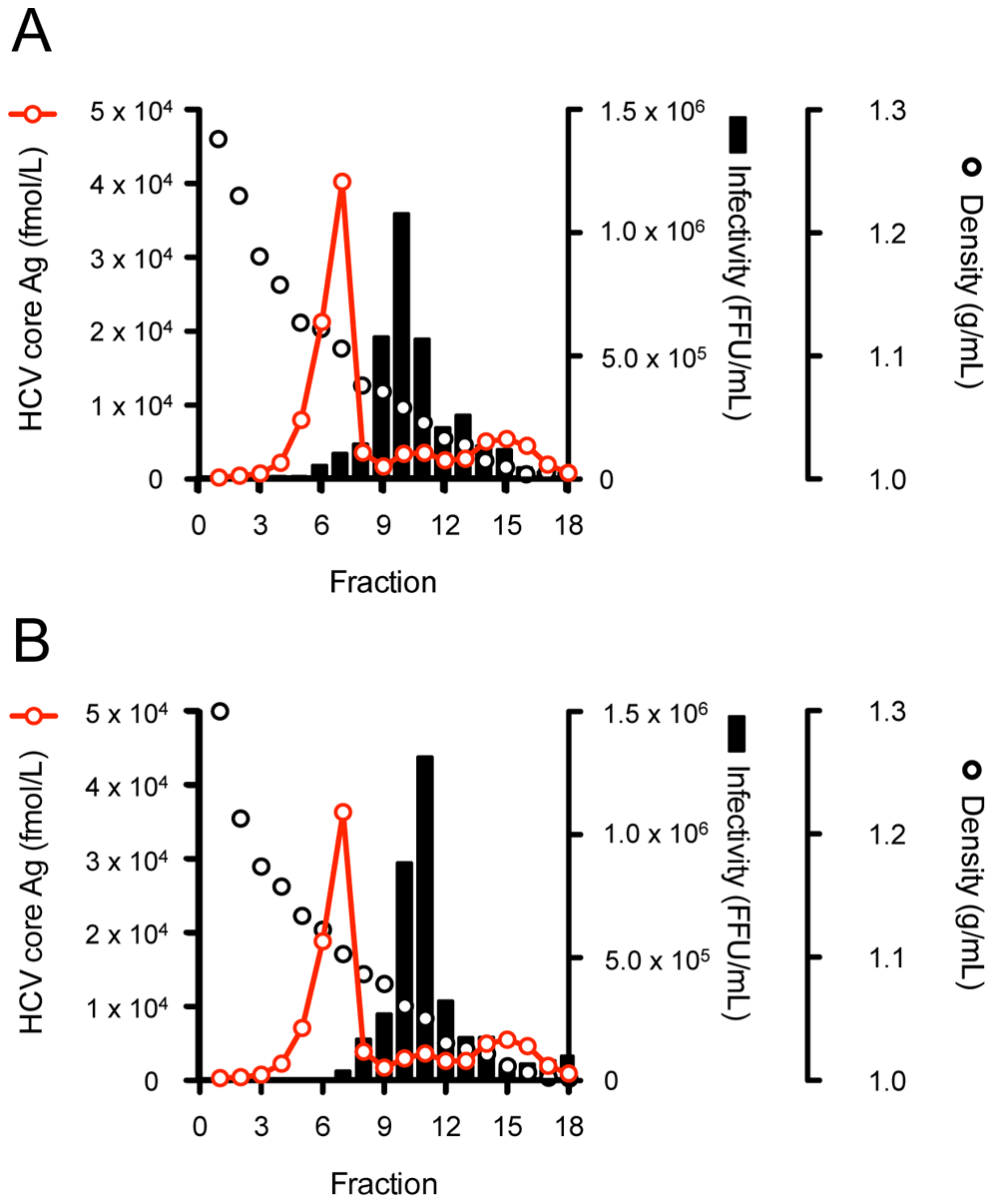
Iodixanol density gradient analysis of JFH-1/2G and JFH-1/6B. Huh7.5.1 cells were transfected with full-length RNAs of JFH-1/2G and JFH-1/6B. Culture medium of each strain was collected and analyzed by 10%–40% of iodixinol density gradient. Fractions were collected, and HCV core Ag and infectivity titers of JFH-1/2G (A) and JFH-1/6B (B) were measured.

Next, specific infectivities were compared. Consistent with previous data, JFH-1/3mut showed enhanced intra- and extra-cellular specific infectivity, as compared with JFH-1/wt, and was approximately 8-fold and 2-fold, respectively (Figure 8B). The strains JFH-1/2G and JFH-1/6B also indicated enhanced intra-cellular specific infectivity, 27.8- and 43.7-fold higher when compared with JFH-1/wt, respectively (Figure 8A). These enhancements were much higher than in the case of JFH-1/3mut. These strains also showed enhanced extra-cellular specific infectivity (4.92- and 5.83-fold, respectively). To assess the biophysical properties of particles with strains of JFH-1/2G and JFH-1/6B, we analyzed the culture medium of these strains transfected cells in the density gradient (Figure 9). The density gradient profiles of these strains were similar to that of JFH-1/wt. The peak densities of infectivity titer of JFH-1/2G (1.06 g/mL) and JFH-1/6B (1.05 g/mL) were almost identical with that of JFH-1/wt (1.07 g/mL), but the peak infectivity titers of these strains were 3 log-fold higher than that of JFH-1/wt.

## Discussion

In this study, we obtained cell culture-adapted virus by serial passage of full-length JFH-1 RNA-transfected Huh-7.5.1 cells for 25 days after transfection. The obtained virus produced 3 log-fold more progeny viruses as compared with JFH-1/wt infected the same amount of FFU. On sequence analysis of this cell culture-adapted virus, 3 amino acid mutations (T416N at E2, K1122R at NS3 and L2525F at NS5B) were identified. On assessment of JFH-1 constructs with these mutations, they were revealed to have advantages in various steps of the viral life cycle. Mutations in K1122R at NS3 and L2525F at NS5B are considered to contribute to efficient infectious virus production. The JFH-1 variants with each mutation, JFH-1/K1122R and JFH-1/L2525F, produced more infectious virus (7.2- and 3.7-fold, respectively), although K1122R slightly reduced intra-cellular viral replication. The mutation T416N at E2 is associated with enhancement of the infection step. JFH-1/T416N showed 2-fold higher efficiency of infection when compared with JFH-1/wt. T416 at E2 is located at the epitope of neutralizing monoclonal antibodies of AP33 and 3/11 and is conserved among genotypes [35]–[37]. The mutation at this site has been detected in cell culture-adapted J6/JFH-1 chimeric virus, but enhanced infectivity has not been observed in the mutation-introduced chimeric virus [20]. Moreover, substitution of T416A was reported to abolish infectivity of HCVpp with envelopes of H77 strain, genotype 1a [38]. In accordance with these data, we found that mutation T416N also reduces the infectivity in the HCVpp system. In contrast, we confirmed that this mutation enhances infectivity in HCVcc and HCVtcp systems. Such discrepancies between HCVpp and HCVcc on the HCV infection step have been reported previously [33]. Because HCVpp is generated in non-hepatic 293T cells, it is likely that the cell-derived components of HCVpp are different from those of HCVcc and HCVtcp. Thus, we believe that the HCVpp system does not reflect the characteristics of mutations in HCV envelopes and may not be suitable for assessing the effects of mutations in the HCV infection step. On the other hand, the T416N mutation in the HCVtcp system indicated consistent data with HCVcc. Therefore, we conclude that this mutation enhances the infectivity of HCV.

The variant JFH-1/3mut with all three mutations, T416N, K1122R and L2525F, has advantages in infectivity and infectious virus production, and results in the highest efficiency of progeny virus production. However, in the infection study, this JFH-1/3mut could not reach the virus production level of the obtained cell culture-adapted JFH-1 virus, JFH-1/day25. We were puzzled by this, and speculated that JFH-1/day25 was not monoclonal, as the direct sequence method is unable to identify responsible mutations associated mixed viruses. Thus, we exploited another strategy. We isolated a single JFH-1 variant that can produce more progeny virus, and we used a method for single virus isolation by infection with a diluted mixture of cell culture-adapted virus. By infection with cell culture-adapted virus at a concentration of 1 FFU/well, we were able to isolate two variants that showed the highest virus production among the tested strains.

The isolated variants, 2G and 6B strains, have additional mutations at p7, NS3 and NS5A. A reverse-genetics analysis revealed that these variants could produce progeny virus more efficiently than JFH-1/wt and JFH-1/3mut after transfection with full-length RNA. In the infection study, the production of progeny virus of these variants was also superior to the levels of JFH-1/wt and JFH-1/3mut, and was comparable to JFH-1/day25. In order to identify the advantages of these variants in the virus life cycle, we used the single cycle virus production assay. JFH-1/2G was able to replicate 1.35-fold more efficiently in culture cells. Both strains have advantages in the steps of infectious virus production and infection. The intra-cellular specific infectivity of JFH-1/2G and JFH-1/6B was 27.8- and 43.7-fold higher, and the extra-cellular specific infectivity was 4.92- and 5.83-fold higher than that of JFH-1/wt. This suggests that enhancement of infectious virus production is a major advantage in these strains. These strains included the additional adaptive mutation V2440L. We examined this sequence in JFH-1/day25 retrospectively, and found a mixture of nucleotide G/C at nucleotide 7658 (data not shown). Thus, there may be many strains with mutations other than V2440L and they are able to propagate efficiently as like as clones, JFH-1/2G and 6B, but we might not be able to isolate such strains in this experiment. This mutation, V2440L, has already been reported in cell culture-adapted JFH-1 virus, and to contribute to slow cleavage at the NS5A-NS5B site, increasing the production of infectious virus [17]. The ability of efficient virus production of JFH-1/2G and JFH-1/6B may be attributable to this mutation.

In conclusion, we were able to successfully isolate 2 cell culture-adapted variants that can produce 3 log-fold more progeny viruses than JFH-1/wt, and identified the responsible mutations. The strategy of single virus isolation by end-point dilution and infection used in this study may be useful for identifying strains with unique characteristics, such as robust virus production, from diverse populations, and for identifying the responsible mutations for these characteristics.
